# Changes in salivary matrix metalloproteinase-3, -8, and -9 concentrations after 6 weeks of non-surgical periodontal therapy

**DOI:** 10.1186/s12903-022-02185-3

**Published:** 2022-05-13

**Authors:** Han-Na Kim

**Affiliations:** Department of Dental Hygiene, College of Health and Medical Sciences, Cheongju University, Cheonju, 28503 Korea

**Keywords:** MMP, Non-surgical periodontal therapy, Oral biomarker, Scaling and root planning, Periodontal disease, Tumor necrosis factor-alpha, Gingival crevicular fluid

## Abstract

**Background:**

Studies using salivary inflammatory biomarkers for diagnosing and monitoring the progression of periodontal disease have garnered increased attention in recent years. The present study aimed to identify changes in clinical parameters and concentrations of salivary matrix metalloproteinases (MMPs) following 6 weeks of non-surgical periodontal therapy (NSPT).

**Methods:**

A 6-week NSPT program was applied to 51 adults aged ≥ 20 years. The program involved scaling, root planing, and professional toothbrushing for healthy participants and those with periodontal disease. Patients with periodontal disease underwent professional toothbrushing during all three visits. Periodontal pocket depth (PD) and gingival bleeding were assessed at week 0, week 3, and week 6, and saliva samples were collected to measure the concentrations of MMP-3, -8, and -9.

**Results:**

All clinical parameters were improved in the periodontal disease groups following the NSPT course. Compared with healthy participants, the patients with periodontal disease showed increased concentrations of salivary MMP-3, -8, and -9. During the 6-week program, patients with periodontal disease also showed significant reductions in PD and gingival bleeding during the third week; no significant reduction was found during the sixth week. Significant reductions in the concentrations of salivary MMP-3, -8, and -9 were also noted in the periodontal disease group at week 3. The sensitivity and specificity of MMP-3 for predicting periodontitis were 81.8% and 55.5%, respectively.

**Conclusion:**

The present study found that NSPT resulted in reductions of salivary MMP-3, -8, and -9, and identified the potential of MMP-3 as a biomarker in the diagnosis of periodontal disease. These findings may serve as foundational data for future studies into the development of diagnostic kits for periodontal disease.

## Background

Periodontal disease occurs mainly in adults, and primarily in those aged ≥ 30 years [1]. The prevalence of periodontal disease is known to increase with age in adults aged ≥ 65 years [2]. Periodontal disease is divided into two phases. The initial phase is characterized by gingivitis that is characterized by gingival swelling, redness, and bleeding. As the condition progresses to the second phase, periodontitis, the inflammation spreads to the periodontal tissues, involving the periodontal ligament and alveolar bone [3]. The progression of periodontitis can cause significant periodontal tissue damage, making recovery of damaged tissues difficult. The disease may also become chronic, further complicating the potential for successful treatment [4, 5]. Therefore, it is imperative to detect periodontal disease early, in order to initiate preventive treatment or employ management strategies to prevent further progression.

Several studies have recently reported on changes in inflammatory biomarkers in the saliva or gingival crevicular fluid (GCF) of patients with periodontal disease, and on the use of these biomarkers in disease diagnosis and treatment [6–9]. Various salivary biomarkers have been proposed as candidates for the diagnosis of periodontal disease [10]. Periodontopathic bacteria, inflammatory mediators, and other causative factors of periodontal disease have been previously identified in the saliva [11]. Of these, matrix metalloproteinases (MMPs) were found at higher concentrations in patients with chronic or advanced periodontal disease when compared with healthy controls [12, 13]. MMPs are a family of enzymes responsible for the degradation of extracellular matrix components such as collagen, proteoglycans, laminin, elastin, and fibronectin [14], and their activity is strictly regulated by the tissue inhibitors of metalloproteinases [15]. The extracellular matrix is thus maintained by an equilibrium in the rates of synthesis, degradation, and connective tissue cell division [16]. During periodontitis, an imbalance of activated MMPs and their inhibitors leads to pathologic breakdown of the extracellular matrix. Because MMP-3 plays a significant role in the activation cascade of latent pro-MMP-1, pro-MMP-8, and pro-MMP-9 [16], its role in periodontal matrix breakdown warrants further investigation.

A recent systematic literature review reported on the satisfactory effects of non-surgical therapy for the management of chronic periodontitis [17]. The effects of non-surgical periodontal therapy (NSPT) have been confirmed by a decrease in periodontal pocket depth (PD) or gingival bleeding [18, 19]. While previous studies have attempted to confirm the therapeutic effects of NSPT based on changes in the concentrations of salivary biomarkers [20, 21], some studies assessed only a few biomarkers, while others reported inconsistent results [22, 23]. Therefore, additional research is needed. Moreover, investigations into the association between salivary biomarkers and periodontal disease progression may serve as a foundation for further research regarding their diagnostic potential.

The purpose of the present study was to evaluate the concentrations of salivary MMPs during a 6-week course of NSPT. This study also aimed to investigate disease progression in participants with and without periodontal disease, based on clinical parameters and changes in salivary MMP concentrations. The hypotheses of this study are as follows: There is no association between MMPs and periodontal disease, and that there will be no effect on clinical parameters of periodontal disease after a six-week NSPT program.

## Materials and methods

### Study population

The study population consisted of 51 medically healthy participants who consented to participation in the NSPT program, conducted between January and February 2020 by the dental hygiene department of a college located in Cheongju, South Korea. All participants were entered the program voluntarily. The participants were divided into a periodontal disease group and a healthy control group based on oral examination results, and all underwent a 6-week NSPT program. Patient periodontal status was classified according to the community periodontal index (CPI) [24]. The present study was conducted with approval from the Cheongju University Institutional Review Board (IRB No. 1041104-201910-BR-036-01).

### Inclusion and exclusion criteria

The inclusion criteria were age ≥ 20 years and an interest in periodontal disease treatment, and who had not received scaling or periodontal treatment during the past six months. The exclusion criteria were loss of more than one third of the posterior teeth, concurrent antibiotic treatment, orthodontic devices or other conditions, infectious disease, oral inflammation or severe gingival bleeding, and scaling or periodontal treatment within the past 6 months.

### Methods

Clinical examinations of all patients were performed three times: at week 0 directly before NSPT treatment (baseline), at week 3, and at week 6. NSPT treatment consisted of either scaling and root planing (SRP) alone, or SRP with professional toothbrushing. Age, sex, and smoking status were recorded for each participant. An oral examination was performed to assess periodontal disease status by dental hygienist (HN Kim), and salivary MMP concentrations were measured using stimulated saliva samples.

#### PD and CAL measurements and periodontal status classification

A calibration-certified dental hygienist examined each periodontal pocket and assessed PD using a PCP UNC 15 periodontal probe (Hu-Friedy, Chicago, IL, USA). CAL was measured and recorded as along with PD measurement. Participants’ periodontal disease status was then assessed using the CPI, as described by the World Health Organization (WHO) [15, 24]. The CPI scores were as follows: normal (CPI 0), gingival bleeding (CPI 1), calculus (CPI 2), shallow periodontal pocket of 3.5–5.5 mm (CPI 3), and deep periodontal pocket of ≥ 5.5 mm (CPI 4).

The CPI probe was used to measure the periodontal PD at six sites per tooth: mesiobuccal, midbuccal, distobuccal, distolingual, midlingual and mesiolingual. Ten teeth were selected for the periodontal examination: two molars in each posterior sextant, and the upper right and lower left central incisors. If an index tooth was absent in a qualifying sextant, the adjacent remaining teeth in that sextant were examined. The highest resulting score was recorded as the final CPI score for each individual. Study groups were then formed according to periodontal status: non-periodontitis (CPI 0, CP 1, and CPI 2) versus periodontitis (CPI 3 and CPI 4).

In the 2018 Classification of Periodontal Diseases, Bhatia et al. [25] reported that the previous classification term “biological width” has since been replaced by “supracrestal tissue attachment”, composed of junctional epithelium and supracrestal connective tissue. The new periodontitis diagnostic criteria can thus be divided into the following four stages [25].

- Stage 1: Initial periodontitis: a very incipient periodontitis with clinical attachment loss and bone loss limited to the most coronal portion of the root.
- Stage 2: Moderate periodontitis: periodontal destruction affecting coronal third of the root and characterized by presence of moderate periodontal pockets (≤ 5 mm).
- Stage 3: Periodontitis with potential for additional tooth loss: presence of furcation and infrabony lesions requiring surgical intervention.
- Stage 4: Severe periodontitis with potential for loss of dentition: presence of masticatory dysfunction and loss of more than five teeth.

Using these criteria, the healthy group in this study included participants without periodontal disease and those with initial periodontitis (stage 1), while the periodontal disease group included those with moderate periodontitis (stage 2) and periodontitis with the potential for additional tooth loss (stage 3).

#### Gingival bleeding measurement

Gingival bleeding was assessed in all teeth excepting the third molar and measured at each visit. After measurement of PD, 1 point was assigned if bleeding was noted in the measured area, and 0 points were assigned if there was no bleeding. The percentage was calculated by dividing the number of bleeding teeth by the total number of teeth examined. Although PD of all teeth was assessed, in accordance with CPI, the exact PD values were recorded for only two molars in each posterior sextant, and for the upper right and lower left central incisors.

#### NSPT protocol

**Fig. 1:**
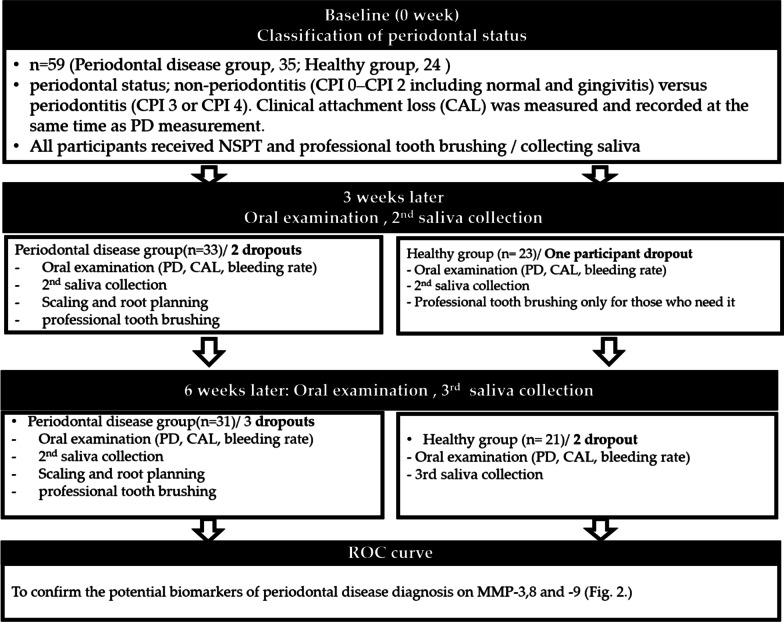
Study flow

Figure 1 showed the flow of NSPT. NSPT protocol included SRP, an oral hygiene education program, and a periodontal health examination. At the baseline visit, all participants underwent mouthrinsing with a 0.12% chlorhexidine mouthrinse (Hexamedine®, Bukgang Medicine, Korea) before receiving SRP treatment and professional toothbrushing with a soft-bristled toothbrush, a compact-tuft toothbrush, an interdental brush, and dental floss (Skydent® oral hygiene education set, Korea). Plaque scores for participants in the periodontitis group were reviewed at 3-week intervals to achieve a score 20% or less. Treatment for the periodontitis group included full mouth debridement with SRP using an ultrasonic scaler (Piezon®, EMS, Nyon, Switzerland) and Gracey curettes (Hu-Friedy, Chicago, IL, USA). Treatment time ranged from 45 to 60 min for each visit. At the initial visit, all participants received a regular tooth brush, toothpaste, interdental brush, and end-tufted brush according to their oral status for use at home, as well as education on oral hygiene. The participants in the periodontal disease group were treated with an interdental brush during the third- and sixth-week visits, and underwent professional toothbrushing and partial scaling as needed. All healthy participants underwent professional toothbrushing during the third-week visit, while only participants with no reduction in gingival bleeding underwent professional toothbrushing during the sixth-week visit. Following completion of the 6-week NSPT course, all participants were reevaluated and additional salivary samples were obtained (Fig. 1).

#### Saliva sampling

For saliva sampling, the participants were instructed to not to eat anything or brush their teeth for at least one hour prior to each visit. Each participant completed a consent form before saliva sampling, and used a new tooth brush supplied by the researcher without toothpaste for one minute, to reduce the amount of oral microorganisms prior to sample collection. For saliva sampling, participants were instructed to chew on paraffin wax (Pinnacle bite modeling wax, Dentsply Sirona, New Zealand), and then a minimum of 2 ml of stimulated saliva was collected in a 10-ml conical tube. Collected saliva was centrifuged (Micro Refrigerated Centrifuge, Micro 17R, Hanil Science, Korea) at 10,000 rpm and 4 °C for 5 min. The separated supernatant was stored in a − 70 °C deep freezer (Ultra Low Temperature Freezer, WUF-500, Daihan Scientific, Korea) until it was analyzed [26].

#### Analytical method for salivary MMPs

Samples were analyzed after thawing in a water bath at 37 °C. We used a 20 μL samples for analyzing MMP concentrations. The MMP concentrations were quantified in units of pg/mL using a multiplexed bead immunoassay (Luminex, Austin, TX, USA) and MAGPIX (Luminex Performance Human XL Cytokine Discovery Magnetic Panel, R&D systems, Minneapolis, MN, USA) according to the manufacturers’ instructions. Results were analyzed using Bio-Plex (Bio-Rad, Hercules, CA, USA).

### Statistical analysis

We evaluated the association between MMPs (dependent variable) and periodontitis, and their coexistence (independent variable). MMP concentrations were used as continuous data, and the presence of periodontitis and smoking status were used as categorical data. MMP values were not normally distributed, thus we performed data transformation for a normal distribution using a natural logarithm (Ln). Log-transformed MMP (Ln MMP) values were normally distributed, with the exception of Ln MMP-3 (Ln MMP-8 *p*-value = 0.901; Ln MMP-9 *p*-value = 0.701; Ln MMP-3 *p*-value < 0.001 using the Kolmogorov–Smirnov test). For statistical analysis, Ln MMP values were used instead of the real values.

To investigate the effects of NSPT therapy, week 0 was set as the baseline and was compared to weeks 3 and 6. The Mann–Whitney U test was performed before and after comparison. In addition, the Wilcoxon test was performed for the comparison of clinical parameters and the Ln MMP concentrations for both patient groups. The confounders for adjustment were age and smoking status. The distribution of the Ln MMP values with respect to confounding variables was expressed as the mean ± standard error (SE). The levels of Ln MMP-3, -8 and -9 for detecting periodontal disease were calculated for sensitivity, specificity, positive predictive value, negative predictive value, and the area under the receiver operating characteristics (ROC) curve. The optimal combination of the parameters for periodontitis screening was determined by the maximum sensitivity and specificity. The significance level was *p* < 0.05.

## Results

### General characteristics of the participants

The initial study population included 59 participants, but the final study population was 51 participants following three dropouts at week 3, and five dropouts at week 6. The mean age was 49 years for participants with periodontal disease and 39 years for participants without periodontal disease, showing a higher mean age for patients with periodontal disease (*p* < 0.001). Study groups with and without periodontal disease had a higher percentage of males (*p* = 0.002). Additionally, the percentage of non-smokers was 58.06% among patients with periodontal disease, and 75% among the healthy controls (*p* = 0.522) (Table 1).

**Table 1:** Characteristics of participants

Variable	Periodontitis		*p*-value
	Y	N	
Age (mean ± SD)	49.52 ± 13.7	39.58 ± 9.8	< 0.001
Sex			
Male, n (%)	19 (61.30)	11 (52.38)	0.002
Female, n (%)	12 (38.70)	9 (47.62)	
Smoking			
Yes, n (%)	13 (41.93)	5 (25%)	0.522
No, n (%)	18 (58.06)	15 (75%)	

Table 2 shows the clinical parameters of the participants. Baseline PD was significantly higher for the patients with periodontal disease (4.07 mm) compared with healthy participants (3.16 mm; *p* < 0.001). The baseline CAL was also significantly higher for patients with periodontal disease (4.87 mm) compared with healthy participants (3.71 mm; *p* < 0.001). Furthermore, baseline gingival bleeding was significantly greater among patients with periodontal disease (67%) compared with healthy participants (29%; *p* < 0.001).

**Table 2:** Differences in clinical parameters

Parameter	Periodontal status	Baseline	Week 3	Baseline to week 3		Week 6	Baseline to week 6	
				Difference	*p*-value*		Difference	*p*-value*
PD (mm)	Periodontitis	4.07 ± 2.35	3.67 ± 1.68	0.4 ± 1.78	**< 0.001**	3.13 ± 1.66	0.94 ± 1.16	0.064
	Health participants	3.16 ± 1.98	3.02 ± 1.49	0.14 ± 1.33	**0.044**	2.98 ± 1.78	0.18 ± 0.98	0.079
	*p*-value^†^	**< 0.001**	**< 0.001**			**< 0.001**		
CAL (mm)	Periodontitis	4.89 ± 2.88	4.32 ± 1.90	0.57 ± 1.15	**0.048**	3.99 ± 2.78	0.99 ± 1.58	**0.040**
	Health participants	3.71 ± 2.56	2.92 ± 1.32	0.79 ± 1.42	0.088	2.90 ± 1.78	0.81 ± 1.82	**< 0.001**
	*p*-value^†^	**< 0.001**	**< 0.001**			**< 0.001**		
Gingival bleeding (%)	Periodontitis	67.01 ± 78.32	34.67 ± 55.51	32.34 ± 52.39	**< 0.001**	23.54 ± 45.66	43.47 ± 62.39	**< 0.001**
	Health participants	29.95 ± 51.23	25.45 ± 30.07	4.5 ± 16.98	**< 0.001**	20.90 ± 52.65	9.05 ± 17.66	**< 0.001**
	*p*-value^†^	**< 0.001**	**< 0.001**			**< 0.001**		

PD, periodontal pocket depth; CAL, clinical attachment level

Bold font denotes statistical significance at *p* < 0.05

*Mann–Whitney U test *p* < 0.01

^†^Wilcoxon test *p* < 0.05

There was a significant decrease in PD at week 3 in patients with periodontal disease (4.07 mm vs 3.67 mm; *p* < 0.001), but no significant decrease was identified at week 6. Among the healthy participants, a significant decrease in PD was found at week 3 (3.16 vs 3.02; *p* < 0.001), but no significant change was found at week 6. A significant decrease in CAL was found only among patients with periodontal disease at week 3 (difference = 0.57 mm) and week 6 (difference = 0.99 mm; *p* < 0.05). Gingival bleeding decreased by 32% among patients with periodontal disease, and by 4.5% among healthy participants at week 3 (*p* < 0.001). By comparison, only CAL and gingival bleeding showed different values at baseline and 6 weeks after NSPT course completion (*p* < 0.001).

Table 3 shows the salivary MMP concentrations of the participants. The baseline salivary MMP-3 concentration was significantly higher in patients with periodontal disease (5.68 Ln pg/ml) compared with healthy participants (4.98 Ln pg/ml; *p* = 0.001). The baseline salivary MMP-8 concentration was also higher in patients with periodontal disease than in healthy participants (*p* = 0.041). Moreover, salivary MMP-9 was also detected at a higher concentrations in patients with periodontal disease (9.38 Ln pg/ml) compared with healthy participants (8.50 Ln pg/ml; *p* < 0.001).

**Table 3:** Changes in concentration of salivary MMPs over 6 weeks

Parameter	Periodontal status	Baseline	Week 3	Baseline to week 3		Week 6	Baseline to week 6	
				Difference	*p*-value*		Difference	*p*-value*
MMP-3 (Ln pg/ml)	Periodontitis	5.68 ± 0.73	5.31 ± 0.92	0.41 ± 0.09	**0.001**	5.34 ± 0.86	0.38 ± 0.17	0.893
	Health subjects	4.98 ± 0.02	4.96 ± 0.15	0.02 ± 0.15	0.88	4.98 ± 0.19	0.00 ± 0.21	0.893
	*p*-value^†^	**0.001**	0.099			0.806		
MMP-8 (Ln pg/ml)	Periodontitis	7.98 ± 2.27	7.14 ± 2.44	0.47 ± 1.38	**0.001**	7.77 ± 2.48	0.81 ± 1.13	0.880
	Health subjects	7.05 ± 2.52	7.39 ± 2.26	0.56 ± 0.50	**0.010**	6.97 ± 1.58	0.68 ± 0.70	0.263
	*p*-value^†^	**0.041**	0.089			**0.010**		
MMP-9 (Ln pg/ml)	Periodontitis	9.30 ± 2.38	9.21 ± 2.26	0.43 ± 1.30	**0.044**	9.03 ± 2.20	0.71 ± 1.09	0.900
	Health subjects	8.50 ± 2.40	8.83 ± 2.34	0.68 ± 0.69	0.187	8.01 ± 1.45	0.80 ± 0.97	0.099
	*p*-value^†^	**< 0.001**	0.620			**< 0.001**		

MMP (Ln pg/ml) means ± SE and *p*-values were adjusted for age (continuous) and smoking

PD, periodontal pocket depth; CAL, clinical attachment level

Bold denotes statistical significance at *p* < 0.05

*Denotes statistically difference between periodontitis and healthy subjects by paired t-test

^†^Calculated with the Wilcoxon test

Patients with periodontal disease showed a significant decrease in salivary MMP-3 at week 3 (*p* < 0.05), but no significant change at week 6. Healthy participants showed no significant decrease at week 3 or week 6. Patients with periodontal disease and healthy participants showed a significant decrease in salivary MMP-8 concentration at week 3 (*p* < 0.05); however, neither group showed a significant change in MMP-8 at week 6. Patients with periodontal disease showed a significant decrease in salivary MMP-9 concentrations only at week 3 (*p* < 0.05), while healthy participants showed no significant decrease in MMP-9 at week 3 or week 6.

**Fig. 2:**
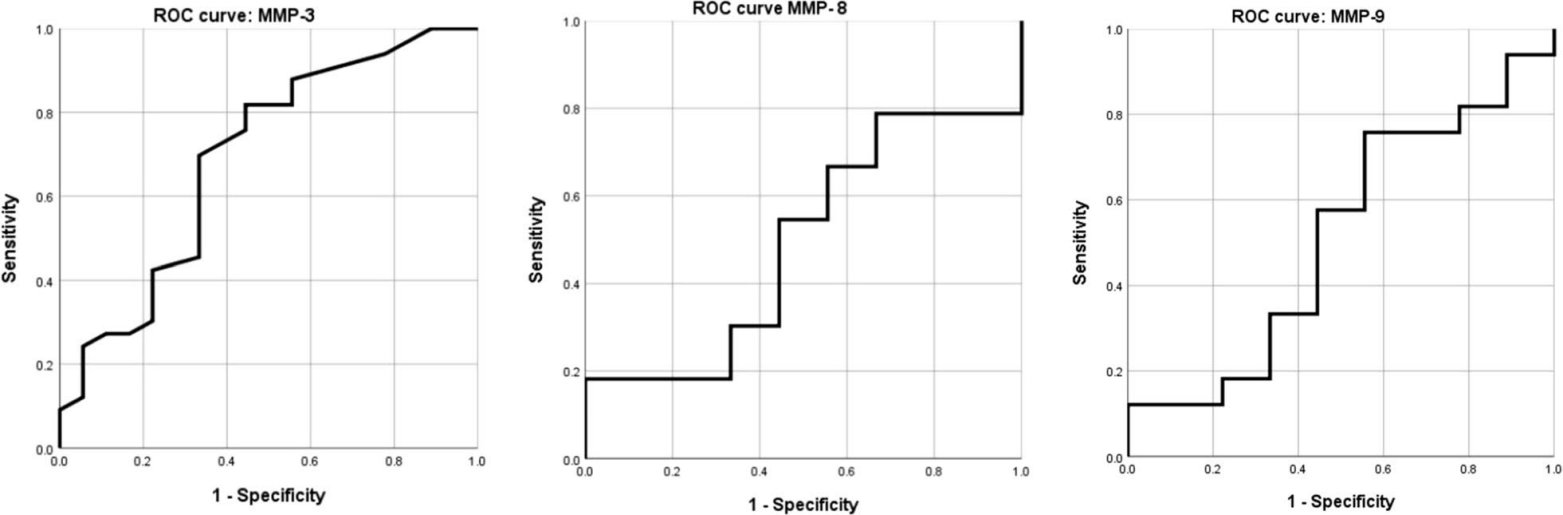
ROC curve of MMP-3,-8 and -9 for periodontal disease diagnosis. Only MMP-3 was a significant salivary biomarker for diagnosis periodontal disease (*p* = 0.024, diagnosis reference value = 5.07 pg/mL)

The predictive factors of MMP-3, -8 and -9 as biomarkers for diagnosis periodontal disease were likewise analyzed with an ROC curve (Fig. 2). Only MMP-3 was found to be a significant biomarker (*p* = 0.024). The threshold value yielding 81.81% sensitivity, 55.5% specificity, 7.71% positive predictive value, and 62.5% negative predictive value for Ln MMP-3 was 5.07 pg/mL. The ROC curves for MMP-8 and MMP-9 indicated they were not significant periodontal disease biomarkers (*p* = 0.921 and *p* = 0.89, respectively).

## Discussion

Periodontal disease is a chronic infectious disease characterized by the destruction of periodontal support tissues, during which the extracellular matrix, particularly collagen, becomes the primary degradation target. The MMP family has been identified as a major factor in this process. MMP-2 and -8 are widely distributed throughout oral tissues; furthermore, MMP-8 expressed by neutrophils is known to affect the progression of inflammation, as well as various inflammatory cells [27]. Increased expression of MMP-2 and -8 in periodontal disease has already been reported by previous studies that analyzed gingival crevicular fluid, which also identified their diagnostic potential for periodontal disease [28]. The mechanism of increased expressions of MMP-2 and -8 in periodontal disease is suspected to involve *Porphyromonas gingivalis* and IL-1 [28]. Accordingly, the values were converted by log transformation for the analysis, as reported in a previous study [29].

In addition to MMPs, there are various other pro-inflammatory biomarkers associated with periodontal inflammation. There is elevated expression of pro-inflammatory cytokines such as IL-1α, IL-1β, IL-6, IL-12, and tumor necrosis factor (TNF)-α, and of regulatory cytokines such as IL-4, IL-1 receptor antagonist (RA), IL-10, and induced protein (IP)-10 [30]. According to Çiğdem et al. [31], TNF-α levels in both GCF and serum decreased with non-surgical periodontal treatment. The progression and extent of tissue degradation is likely determined in part by the relative concentrations and half-lives of IL-1, TNF-α, and related cytokines, as well as competing molecules such as IL-1 RA, and suppressive molecules such as TGF-*β* and PGE2 [32]. There have been many related studies into the roles of biomarkers in periodontal disease [33–35], but few have quantitatively compared the salivary concentrations of said biomarkers before and after NSPT therapy.

In the present study, salivary MMP concentrations were investigated as inflammatory biomarkers of periodontal disease, and the measured concentrations were converted by log transformation for analysis. The study findings showed statistically significant decreases in salivary MMP-3, -8, and -9 concentrations in patients with periodontal disease 3 weeks after NSPT initiation. In another study with similar results, Morelli et al. [36] reported that higher salivary concentrations of MMP-3, -8, and -9 and neutrophil gelatinase-associated lipocalin were found in the diseased groups than in the healthy control group. A meta-analysis by Weng et al. [37] on the association between periodontitis and gene polymorphisms reported that MMP-3 and -8 increased the risk of periodontal disease. A study by Görgülü et al. [38] involving non-smokers reported that the salivary MMP-8 concentration was higher in patients with periodontal disease than in healthy participants, and the MMP-8 concentration decreased following NSPT. Studies investigating MMPs and various other salivary biomarkers for the diagnosis and identification of disease progression have garnered much attention, and the present study likewise confirmed this potential.

MMP-1 is thought to be a key player in the breakdown of periodontal tissues [39]. Additionally, MMP-3 is known to destroy periodontal tissues near the affected area and stimulate periodontal ligament cells via TNF-α [38]. MMP-3 also has a pivotal role in activating latent MMPs including pro-MMP-1, -8 and -9 [40]. Therefore, MMP-3 is an integral element in the destruction of periodontal connective tissues.

Periodontitis is a polymicrobial and multifactorial disease for which clinical measures, such as PD, CAL, and bleeding on probing (BOP), provide insight into disease history and current status. The present study investigated the effects of a 6-week NSPT program on these three parameters. Three weeks following initiation of NSPT, there was a significant decrease in PD, CAL, and gingival bleeding in patients with periodontal disease. Cob et al. [41] similarly reported probing depth reduction and clinical attachment gain after NSPT, thus our findings are in agreement.

NSRP has some disadvantages as well. Root sensitivity occurs in approximately half of patients after treatment. The sensitivity can increase for several weeks following therapy before it gradually decreases [42]. Smoking and uncontrolled diabetes may limit the success of NSPT. Accordingly, the 6-week program used in the present study focused on reducing gingival bleeding by providing gingival massaging to the participants throughout the program. In the present study, the 6-week NSPT program was applied to two different groups: patients with periodontal disease and healthy subjects. All participants underwent NSRP, followed by professional toothbrushing using a regular tooth brush and an interdental brush. During the professional toothbrushing, a dentist or a dental hygienist brushed the patient’s teeth. Brushing was then followed by a systematic removal of plaque and application of gingival massage, which has been proven to provide preventive benefits by promoting bacterial resistance and heightening the immunity of periodontal tissues [43]. Many studies have reported that professional toothbrushing can reduce gingival bleeding and dental plaque [44, 45]. Moreover, interdental brushes have been shown to be the most effective interdental cleaning aids. A systematic review by Slot et al. [46] demonstrated a significant reduction in dental plaque, BOP and PD values following the use of interdental brushes. In the present study, patients with periodontal disease underwent deep scaling to remove dental calculus. At the week 6 assessment, the periodontal pocket was also cleaned as the subgingival space became accessible after a reduction in gingival inflammation. Therefore, professional toothbrushing and NSRP may have contributed to the decrease in salivary MMP concentrations; however, it is difficult to determine the individual effect of each treatments.

The ROC curve showed that of the three MMPs analyzed, the only significant biomarker was MMP-3 (*p* = 0.024), as confirmed through sensitivity and specificity analysis. Although not identical biomarkers, Sanchez et al. [47] reported that with a selected threshold of 212 pg/mL, salivary IL-1β predicted periodontitis with 78% sensitivity and 100% specificity. It is challenging to suggest an absolute value of MMP-3 for predicting periodontitis, as the value of making a clinical diagnosis using an ROC curve varies depending upon the measurement environment and target.

A recent systematic review evaluated macrophage inflammatory protein-1α (MIP-1α), IL-1β, IL-6 and MMP-8 as diagnostically acceptable biomarkers for periodontal disease, and the combination of IL-6 and MMP-8 showed the best diagnostic performance [48]. Another systematic review by de Lima et al. [49] suggested that one biomarker in particular, MIP-1α, had excellent diagnostic accuracy while two others, IL-1β and IL-6, showed acceptable diagnostic accuracy. Despite the results of such systematic literature reviews, continuous research is still necessary because the number of research participants and related studies is thus far insufficient.

The present study had several limitations. First, the recruited participants were fewer than expected due to the COVID-19 pandemic, and a sufficient sample size could not be secured. Consequently, the study population had a very broad age range, resulting in varying degrees of periodontal disease progression. To compensate for this, age and sex were adjusted when comparing the mean values. Second, there were many smokers among the periodontal disease patients, which may have affected salivary samples. Third, while most studies have monitored the progression of periodontal disease and therapeutic effects over 6 months [50, 51], the present study measured the effects of NSPT after 6 weeks; as a result, sufficient therapeutic effect and long-term changes could not be identified. Future studies should observe changes in a larger population over at least a 6-month period. Moreover, because periodontal disease affects and is affected by other systemic diseases, it is also necessary to increase the sample size to consider related diseases and socioeconomic factors [52, 53]. Fourth, in this study the CPI index was applied for the diagnosis of periodontal disease, the criterion for diagnosing periodontal disease is that classification term of biological width is replaced by supracrestal tissue attachment, consisting of junctional epithelium and supracrestal connective tissue [25]. As the CPI index was used to diagnosis of periodontal disease, I also presented along with the new periodontal disease diagnosis criteria to enhance the reader's understanding. Lastly, probing at 3-week intervals would not have had a positive effect on periodontal tissue healing. Periodontal soft tissue wound healing and supracrestal tissue attachment formation requires approximately 6 to 8 weeks [54]. Therefore, when it was necessary to assess gingival bleeding on all teeth, an effort was made to probe as gently as possible.

## Conclusion

While many previous studies have investigated salivary biomarkers of periodontal disease, few have assessed the change in biomarkers before and after NSPT therapy. The present study assessed the therapeutic effects of a 6-week NSPT course by measuring clinical parameters and salivary MMP concentrations in two population groups. The results showed that salivary MMP-3, -8, and -9 decreased significantly 3 weeks after NSPT initiation, while clinical parameters improved. Compared with healthy participants at baseline, the periodontal disease group also showed higher salivary MMP-3, -8, and -9 concentrations. MMP-3 as a biomarker for the diagnosis of periodontal disease was identified, with a predictive sensitivity and specificity of 81.8% and 55.5%, respectively. The potential application of saliva-based testing for periodontal disease represents an exciting new opportunity in noninvasive chairside diagnostics. In this respect, the present study could form the groundwork for further exploration into the diagnostic potential of salivary biomarkers, and ultimately the development of diagnostic kits for periodontal disease.

## Data Availability

The data that support the findings of this study are available from the National Research Foundation (NRF) funded by the Ministry of Science and ICT (MSIT), but restrictions apply to the availability of these data, which were used under license for the current study and so are not publicly available. Data are however available from the author upon reasonable request.

## Abbreviations

BOP: Bleeding on probing
CAL: Clinical attachment level
CPI: Community periodontal index
GCF: Gingival crevicular fluid
Ln MMPs: Log-transformed values of matrix metalloproteinases
MIP-1α: Macrophage inflammatory protein-1αlpha
MMP: Matrix metalloproteinase
NSPT: Non-surgical periodontal therapy
PD: Pocket depth
SRP: Scaling and root planing
TNF-α: Tumor necrosis factor-alpha
